# Nipah virus vaccines evaluated in pigs as a ‘One Health’ approach to protect public health

**DOI:** 10.1038/s41541-025-01212-y

**Published:** 2025-07-23

**Authors:** Rebecca K. McLean, Miriam Pedrera, Nazia Thakur, Ahmed M. E. Elrefaey, Sophia Hodgson, Sue Lowther, Tristan Reid, Shawn Todd, Brenton Rowe, Jemma Bergfeld, Lee Trinidad, Sarah Riddell, Sarah Edwards, Jean Payne, Jennifer Barr, Nick Rye, Matt Bruce, Tim Poole, Sheree Brown, Toni Dalziel, Gough Au, Megan Fisher, Rachel Layton, Teresa Lambe, Keith Chappell, Ariel Isaacs, Daniel Watterson, Mercedes Mourino, Ruediger Raue, Ireen Sultana Shanta, Ayesha Siddika, Mst Noorjahan Begum, Sezanur Rahman, Abdulla Al Mamun Bhuyan, Muntasir Alam, Mohammed Ziaur Rahman, Mustafizur Rahman, Elma Tchilian, Sarah C. Gilbert, Paul Young, Dalan Bailey, Glenn A. Marsh, Simon P. Graham

**Affiliations:** 1 The Pirbright Institute, Pirbright, UK; 2 Australian Centre for Disease Preparedness, Geelong, VIC Australia; 3 Jenner Institute, Nuffield Department of Medicine, University of Oxford, Oxford, UK; 4 School of Chemistry and Molecular Biosciences, University of Queensland, Brisbane, QLD Australia; 5 Zoetis Manufacturing & Research Spain S.L, Girona, Spain; 6 Zoetis, Zaventem, Belgium; 7 Infectious Diseases Division, International Centre for Diarrhoeal Disease Research, Bangladesh, Dhaka, Bangladesh; 8 Department of Veterinary & Animal Sciences, University of Rajshahi, Rajshahi, Bangladesh; 9 Present Address: Centro de Investigación en Sanidad Animal (CISA), Instituto Nacional de Investigación y Tecnología Agraria y Alimentaria (INIA), Consejo Superior de Investigaciones Científicas (CSIC), Valdeolmos, Madrid, Spain; 10 Present Address: Oxford Vaccine Group, Department of Paediatrics, and Chinese Academy of Medical Sciences-Oxford Institute, University of Oxford, Oxford, UK; 11 Present Address: Pandemic Sciences Institute, Nuffield Department of Medicine, University of Oxford, Oxford, UK

**Keywords:** Vaccines, Vaccines, Virology

## Abstract

Nipah virus (NiV) causes a severe neurological disease in humans. The first NiV outbreak, in Malaysia, involved pig-to-human transmission, that resulted in significant economic losses to the local pig industry. Despite the risk NiV poses to pig-dense regions, no licensed vaccines exist. This study therefore assessed three NiV vaccine candidates in pigs: (1) adjuvanted soluble NiV (s)G protein, (2) adjuvanted pre-fusion stabilised NiV (mcs)F protein, and (3) adenoviral vectored NiV G (ChAdOx1 NiV G). NiV sG induced the strongest neutralising antibody response, NiV mcsF induced antibodies best able to neutralise cell-cell fusion, whereas ChAdOx1 NiV G elicited CD8^+^ T-cell responses. Despite differences in immunogenicity, prime-boost immunisation with all candidates conferred a high degree of protection against NiV infection. Follow-up studies demonstrated longevity of immune responses and broadly comparable immune responses in Bangladeshi pigs under field conditions. These studies provide a platform for developing a NiV vaccine for pigs.

## Introduction

The zoonotic Nipah virus (NiV) is a member of the *Henipavirus* genus within the *Paramyxoviridae* family^1^. NiV has garnered significant attention due to its potential to cause severe illness in humans, posing a substantial threat to public health. Originating from Old World fruit bats of the *Pteropodidae* family, which are broadly distributed across South and Southeast Asia, NiV primarily affects pigs and humans^2^. Transmission of NiV occurs through direct contact with infected animals or their bodily fluids. Humans can become infected through close contact with infected pigs, bats or their excretions^3,4^. Furthermore, human-to-human transmission has been documented via respiratory routes and direct contact^5^, particularly in southern India making containment challenging. There are two major clades of NiV: NiV-Malaysia (NiV_M_) and NiV-Bangladesh (NiV_B_) but there are strains which have been isolated from Malaysia, Cambodia, India and Thailand^6,7^. NiV_M_ was first identified during a 1998–1999 outbreak in Malaysia and typically exhibits a lower case-fatality rate of approximately 40%^8^. In contrast, NiV_B_, first recognised in 2001, has a higher case-fatality rate of over 70% and is linked to direct human-human transmission and consumption of contaminated date palm sap^9^. The Indian and Bangladeshi strains cluster closely together based on the nucleocapsid sequence^7^.

Pigs can play a crucial role in the transmission dynamics of NiV, serving as intermediate hosts between bats and humans^10^. Due to a lack of pathognomonic signs of NiV infection and their proximity to humans in agricultural settings, pigs can function as amplifying hosts, facilitating the spread of the virus to humans. The high-density farming practices prevalent in Southeast Asia further exacerbate the risk of transmission within pig populations. The emergence of NiV in Malaysia in the late 1990s had devastating effects on the local pig industry. The outbreak led to massive culling of pigs (approximately 45% of the national pig population) to control the spread of the virus, resulting in substantial economic losses for pig farmers and the agricultural sector as a whole^8^. Addressing the threat of NiV requires a comprehensive ‘One Health’ approach^11^. Collaborative efforts between veterinarians, public health officials, environmental scientists, and policymakers are essential for effective surveillance, prevention, and control of the virus. By integrating expertise from multiple disciplines, strategies can be developed to minimise the risk of spillover events and mitigate the impact of outbreaks on both human and animal populations. Vaccines could play a crucial role in preventing the spread of NiV and mitigating the risk of a future pandemic. Vaccination programs targeted at pigs could reduce the likelihood of further transmission^12^ and limit the impact of outbreaks on public health and the economy.

An adjuvanted soluble subunit vaccine that contains the attachment protein of the closely related Hendra virus (HeV sG) has been licensed in Australia to protect horses against HeV and help reduce the zoonotic risk to humans (Equivac® HeV, Zoetis)^13^. Despite this vaccine protecting both ferrets and African green monkeys, it surprisingly failed to protect pigs against experimental NiV challenge^14^. A canarypox virus (ALVAC strain) vector expressing NiV G or fusion (F) glycoproteins protected pigs against NiV challenge^15^; however, there are currently still no NiV vaccines licensed for use in pigs, despite these animals acting as the amplifying host for the initial outbreak of NiV. A limited number of NiV vaccine candidates are now entering human clinical trials, including a recombinant HeV sG subunit vaccine; recombinant vesicular stomatitis virus expressing NiV_B_ strain G; a chimpanzee adenoviral vector (ChAdOx1) expressing NiV_B_ G, and an mRNA vaccine that encodes the secreted prefusion stabilised F component covalently linked to G monomer of NiV_M_^16^. The pig also provides a valuable preclinical model for testing vaccines before clinical trials in humans, due to the anatomical, physiological, and immunological similarities between these species^17–19^. Thus, the evaluation of the immunogenicity and efficacy of NiV vaccine candidates in pigs would inform the ongoing efforts to develop a human vaccine.

In this study, we assessed the potential of three novel NiV vaccine candidates in pigs. A chimpanzee adenoviral vector encoding NiV_M_ G (ChAdOx1 NiV G)^20,21^; a molecular clamp stabilised NiV_M_ F protein (NiV mcsF)^22^ and a soluble NiV_M_ G protein (NiV sG)^23^ were assessed for immunogenicity in mice and pigs and protective efficacy in pigs (against homologous challenge) before being assessed in ‘backyard’ pigs under field conditions in the ‘Nipah belt’ region of Bangladesh.

## Results

### NiV vaccine candidates are immunogenic in mice

**Fig. 1:**
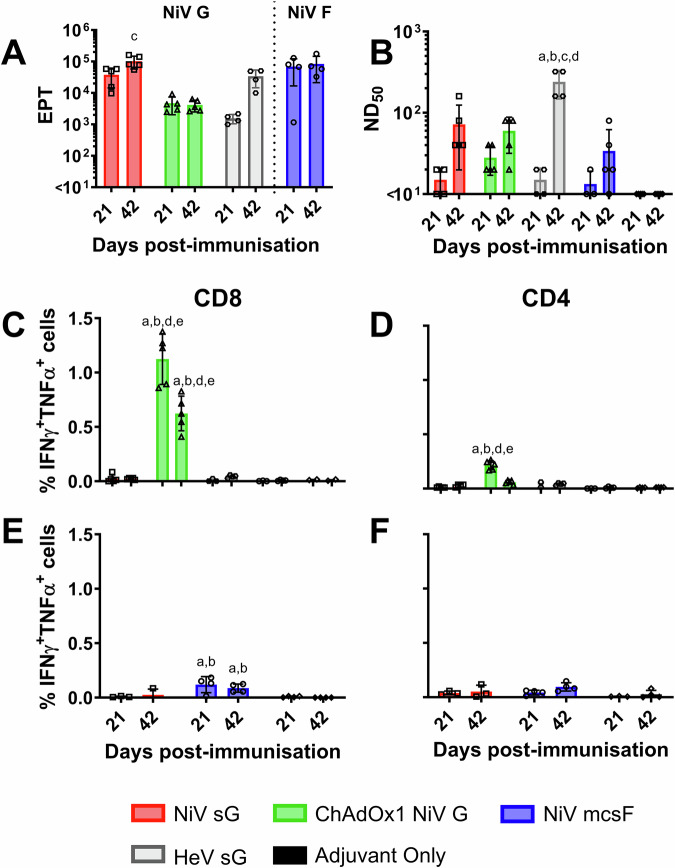
Immunisation of mice with NiV sG, ChAdOx1 NiV G or NiV mcsF induces varied antibody and T-cell responses. Mice were immunised on day 0 and 21 by intramuscular inoculation of 5 µg NiV sG, NiV mcsF, or HeV sG proteins in adjuvant, or 1 × 10^8^ IU ChAdOx1 NiV G. **A** NiV mcsF and sG binding antibody titres (EPT) in serum on day 21 and 42; **B** Virus neutralising titres in day 21 and 42 serum as assessed by NiV_M_ VNT; **C**, **D** CD8^+^ and CD4^+^ T-cell cytokine responses after stimulation of splenocytes with NiV G peptides, respectively; **E**, **F** CD8^+^ and CD4^+^ T-cell cytokine responses after stimulation of splenocytes with NiV F peptides, respectively. Splenocytes from all groups were restimulated with NiV G peptides, whereas splenocytes from the NiV sG, NiV mcsF and adjuvant only groups were restimulated with NiV F peptides. Datapoints represent individual mice, with the bars showing the group mean and error bars represent the standard deviation (SD). Significant differences were determined using two-way ANOVA and signified with the following letter: a—significant difference to adjuvant; b—significant difference to NiV sG; c—significant difference to ChAdOx1 NiV G; d—significant difference to NiV mcsF; e—significant difference to HeV sG.

NiV vaccine candidates (adjuvanted NiV sG protein, adjuvanted NiV mcsF protein, and ChAdOx1 NiV G) were first evaluated for immunogenicity in BALB/c mice. Responses were compared to those elicited by immunisation with adjuvanted HeV sG protein (the antigen used in the licensed equine HeV vaccine). Serum antibody and splenocyte responses were assessed 21 days after prime and booster immunisations (Fig. 1). A single immunisation with all four vaccines induced antigen-binding antibody titres. Immunisation with NiV sG produced significantly higher NiV-G specific antibody titres at day 42 (determined by ELISA) when compared to serum from ChAdOx1 NiV G immunised mice (*p* < 0.01) (Fig. 1A). Immunisation with NiV mcsF induced high titres of F-binding antibodies, comparable to the G-binding titres induced by NiV sG. All vaccines induced NiV neutralising antibody titres after a single immunisation, and titres were elevated following the booster immunisation, with only the HeV sG group showing a significant increase in titres following the boost. Unexpectedly, the highest neutralising titres were observed in day 42 sera from HeV sG immunised mice (Fig. 1B). ChAdOx1 NiV G was the only vaccine to show substantial frequency of CD8^+^ T-cells producing IFN-γ and TNF-α, with a significantly higher frequency observed in the mice culled at day 21 (Fig. 1C). NiV G-specific CD4^+^ T-cell responses were also most prominent in the ChAdOx1 NiV G immunised mice, also with a significant higher frequency observed in the mice culled at day 21, although they were lower than the CD8^+^ T-cells (Fig. 1D). Mice immunised with NiV mcsF showed a significant CD8^+^ T-cell response, although the magnitude was lower than observed in ChAdOx1 NiV G immunised mice (Fig. 1E). The limited impact of booster immunisation on antibody titres and T cell responses were similar to that observed previously in mice immunised with a COVID-19 vaccine candidate^24^, and suggests that the vaccine dosages used may have saturated the immune response and obscured the ability to discern the impact of the boost.

### NiV vaccine candidates are immunogenic in pigs

**Fig. 2:**
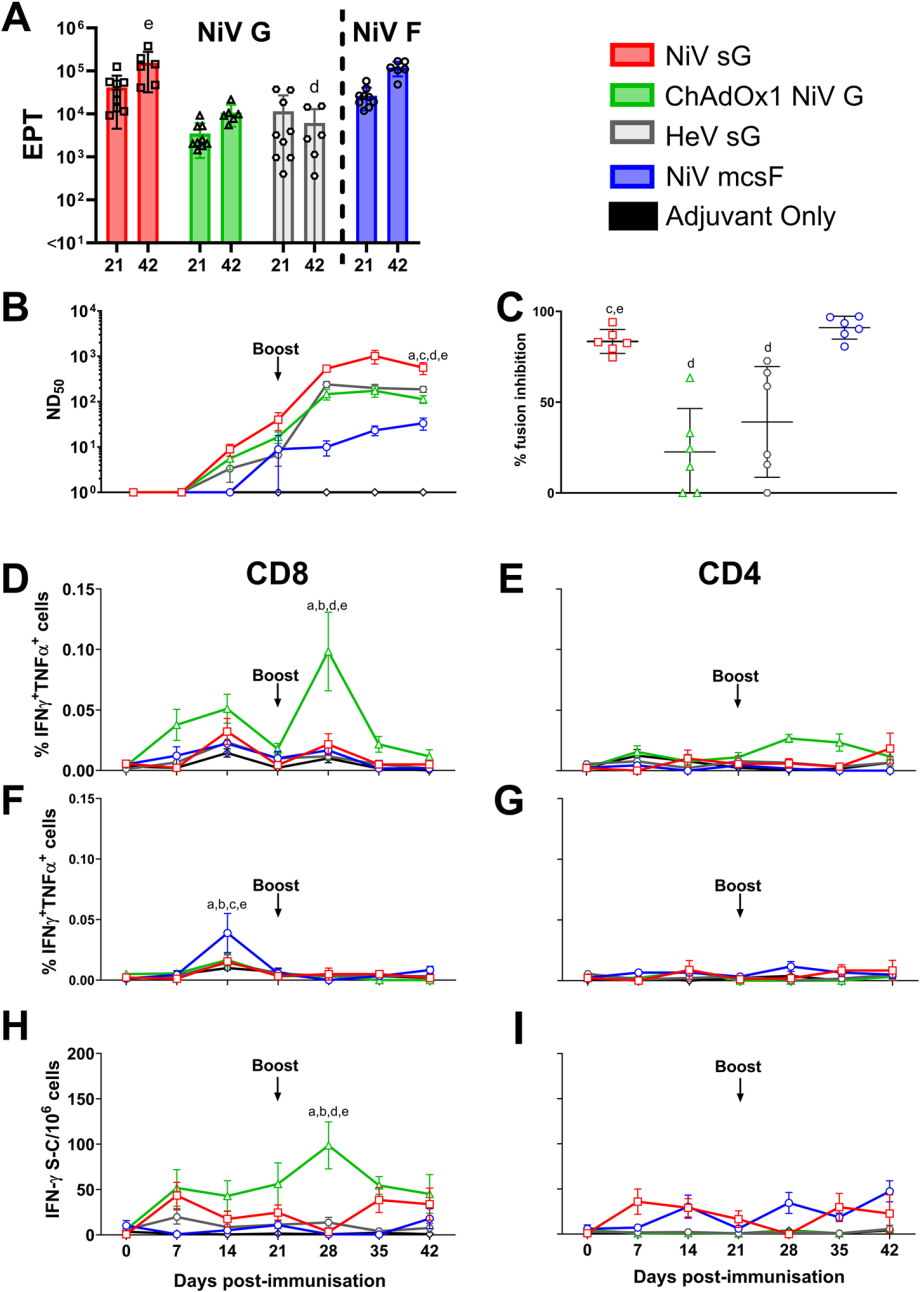
Immunisation of pigs with NiV sG, ChAdOx1 NiV G, NiV mcsF similarly induces varied antibody and T-cell responses. Pigs were immunised on day 0 and 21 by intramuscular inoculation of 100 µg NiV sG, NiV mcsF, or HeV sG proteins in adjuvant, or 1 × 10^9^ IU ChAdOx1 NiV G. **A** NiV mcsF and sG binding antibody titres (EPT) on day 21 and 42; **B** Virus neutralising titres as assessed by NiV_M_ VNT; **C** Inhibition of NiV glycoprotein-mediated cell-cell fusion evaluated using day 42 sera; **D**, **E** CD8^+^ and CD4^+^ T-cell cytokine responses after stimulation of PBMC with NiV G peptide pool, respectively; **F**, **G** CD8^+^ and CD4^+^ T-cell cytokine responses after stimulation of PBMC with NiV F peptide pool, respectively. **H**, **I** IFN-γ ELISpot assay to assess PBMC responses to NiV G and F peptide stimulation, respectively. For **A** and **C**, datapoints represent individual pigs, with the bars showing the group mean and error bars represent the SD. For the other panels, datapoints represent the group mean and error bars represent the SD. Significant differences were determined using two-way ANOVA and signified with the following letter: a—significant difference to adjuvant; b—significant difference to NiV sG; c—significant difference from ChAdOx1 NiV G; d—significant difference to NiV mcsF; e—significant difference to HeV sG.

Since all three vaccine candidates were immunogenic in mice, they were next evaluated for immunogenicity in pigs (Fig. 2). Responses were once again benchmarked against the HeV sG antigen in adjuvant. All vaccines appeared to show no reactogenicity, with no significant elevation in rectal temperatures nor other clinical signs being observed (Supplementary Fig. 1). After receiving a homologous booster immunisation on day 21, a significant increase in NiV G binding antibody titres was detected in day 42 serum from pigs immunised with NiV sG and ChAdOx1 NiV G (*p* < 0.001 and <0.01, respectively) (Fig. 2A). NiV G antibody titres in pigs immunised with NiV sG were significantly higher on day 42 compared to pigs immunised with HeV sG (*p* < 0.0001) (Fig. 2A). The kinetic of this antibody response is shown in Supplementary Fig. 2. All vaccines induced neutralising antibody titres (Fig. 2B). One-week post-boost, there was a significant increase in neutralising titres in NiV sG immunised pigs (*p* < 0.0001), although all vaccines showed a trend for increased titres following the boost. On day 42, pigs immunised with NiV sG had significantly higher neutralising titres compared to all other vaccine candidates (HeV sG *p* < 0.01; ChAdOx1 NiV G *p* < 0.0001 and NiV mcsF *p* < 0.0001). The ability of sera to reduce NiV glycoprotein mediated cell-to-cell fusion was assessed using the mFIT assay (Fig. 2C). Sera from pigs immunised with NiV sG and NiV mcsF inhibited fusion significantly more than pigs immunised with ChAdOx1 NiV G (*p* < 0.0001) and HeV sG (*p* < 0.01 and <0.001, respectively). Like the mouse data, ChAdOx1 NiV G was the only vaccine to show significant production of CD8^+^ T-cells producing IFN-γ and TNF-α at day 28 (7 days post-boost; *p* < 0.0001) (Fig. 2D). T-cells producing single cytokines (IFN-γ or TNF-α) are shown in Supplementary Fig. 3. A moderate increase in CD4^+^ T cells producing IFN-γ and TNF-α was detected after boosting with ChAdOx1 NiV G (Fig. 2E). Pigs immunised with NiV mcsF showed a significant increase in CD8^+^ T-cells producing IFN-γ and TNF-α at day 14 when compared to day 7 (*p* < 0.0001) but no boosting effect was observed (Fig. 2F). No notable NiV F specific CD4^+^ T cell response could be detected (Fig. 2G). The peak in IFN-γ responses at 28 dpv was also shown using an IFN-γ ELISpot assay (Fig. 2H) where, on day 28, the number of IFN-γ secreting cells in PBMC from ChAdOx1 NiV G immunised pigs was significantly higher than all other groups (*p* < 0.0001). Of note, pigs immunised with NiV sG showed spontaneous secretion of both IFN-γ and TNF-α at all timepoints post-immunisation in both the flow cytometric and IFN-γ ELISpot assays, for reasons that could not be elucidated (Supplementary Figs. 4 and 5).

### Prime-boost immunisation of pigs with NiV vaccine candidates significantly reduces virus shedding and loads post-challenge

**Fig. 3:**
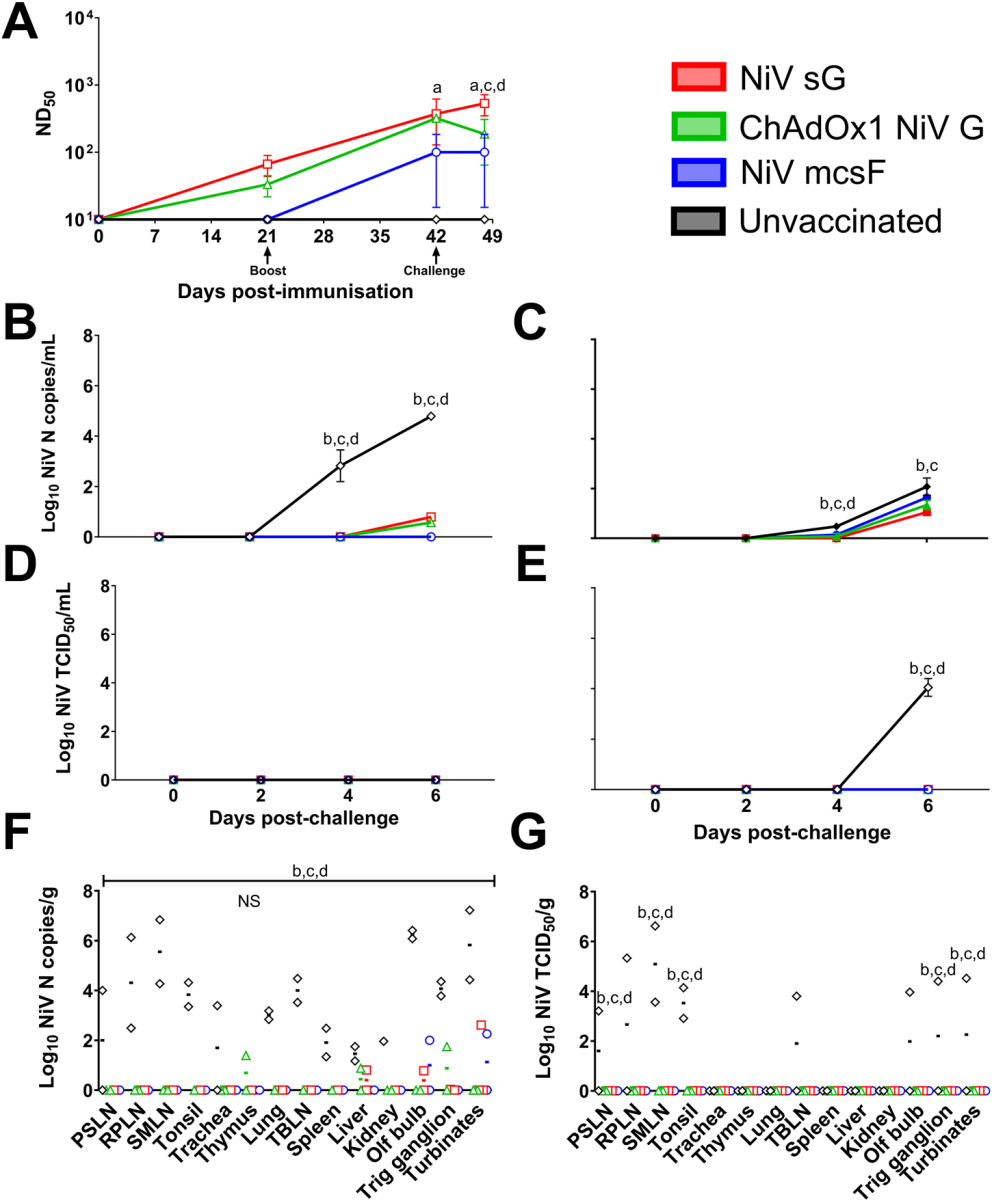
NiV vaccine candidates are protective in pigs following a prime-boost immunisation regimen. Pigs were immunised on day 0 and 21 by intramuscular inoculation of 100 µg NiV sG or NiV mcsF proteins in adjuvant, or 1 × 10^9^ IU ChAdOx1 NiV G. On day 42, all pigs were challenged by oronasal inoculation with 1 × 10^5^ PFU NiV_M_. **A** Neutralising antibody titres as assessed by NiV_M_ VNT; **B**, **C** Viral loads in nasal and oral swabs detected post-challenge by RT-qPCR, respectively. **D**, **E** Level of infectious NiV from nasal and oral swabs detected post-challenge by virus isolation, respectively. **F**, **G** Viral loads from tissue samples at 6 days post-challenge detected by RT-qPCR and virus isolation, respectively. Postmortem tissues collected: PSLN prescapular lymph node, RPLN retropharyngeal lymph node, SMLN submandibular lymph node, TBLN tracheobronchial lymph nodes, Olf bulb olfactory bulb, Trig ganglion trigeminal ganglion. Significant differences were determined using two-way ANOVA and signified with the following letter: a—significant difference to unvaccinated; b—significant difference to NiV sG; c—significant difference from ChAdOx1 NiV G; d—significant difference to NiV mcsF. NS not significant.

After determining immunogenicity profiles in pigs, which were unique to each vaccine administered, three vaccine candidates were evaluated in an efficacy study, using the same prime-boost immunisation regimen (the adjuvanted HeV sG protein was not included in this and subsequent studies). Pigs were then challenged on day 42 by oronasal inoculation of NiV_M_. Since experimental infection of pigs with NiV_M_ did not induce clinical disease, protection was determined by reduction in viral shedding and viral loads in tissues harvested after 6 days; which has been described as the peak of infection^15^ (Fig. 3). Neutralising antibody titres at days 21 and day 42 (Fig. 3A) were comparable to those seen in the previous immunogenicity study (Fig. 2B). At the point of challenge at day 42 post-immunisation, only pigs receiving NiV sG had a significantly higher level of neutralising antibodies to the unvaccinated control pigs. At 6 days post-challenge, pigs immunised with NiV sG had significantly higher neutralising antibodies than those immunised with ChAdOx1 NiV G (*p* < 0.05) and NiV mcsF (*p* < 0.01) and the unvaccinated controls (*p* < 0.001) (Fig. 3A). At 4 days post-challenge, unvaccinated pigs had significantly higher copies of NiV RNA in both nasal (*p* < 0.0001) and oral (*p* < 0.05) swab samples than animals in the three vaccine groups (Fig. 3B, C, respectively). At 6 days post-challenge, significantly lower NiV RNA copies were detected in the nasal swabs of all vaccinated pigs when compared to unvaccinated controls (*p* < 0.0001) (Fig. 3B), whereas only pigs immunised with NiV sG or ChAdOx1 NiV G had significantly lower (*p* < 0.0001) NiV RNA in oral swabs compared to the unvaccinated controls (Fig. 3C). Infectious NiV could only be detected in oral swabs from unvaccinated pigs (*p* < 0.0001) (Fig. 3E). At post-mortem, significantly higher amount of NiV RNA copies were found in all tissues sampled from unvaccinated pigs (*p* < 0.0001), except the thymus, which was not significantly different from the vaccinated pigs (Fig. 3F). Infectious NiV could not be isolated from tissues of vaccinated pigs but could from unvaccinated controls; with prescapular (*p* < 0.01) and submandibular lymph nodes (*p* < 0.0001), tonsil (*p* < 0.0001), trigeminal ganglion (*p* < 0.05) and nasal turbinates (*p* < 0.05) being significantly different from the vaccinated groups (Fig. 3G).

### NiV vaccine candidates are not effective in pigs following a single immunisation

**Fig. 4:**
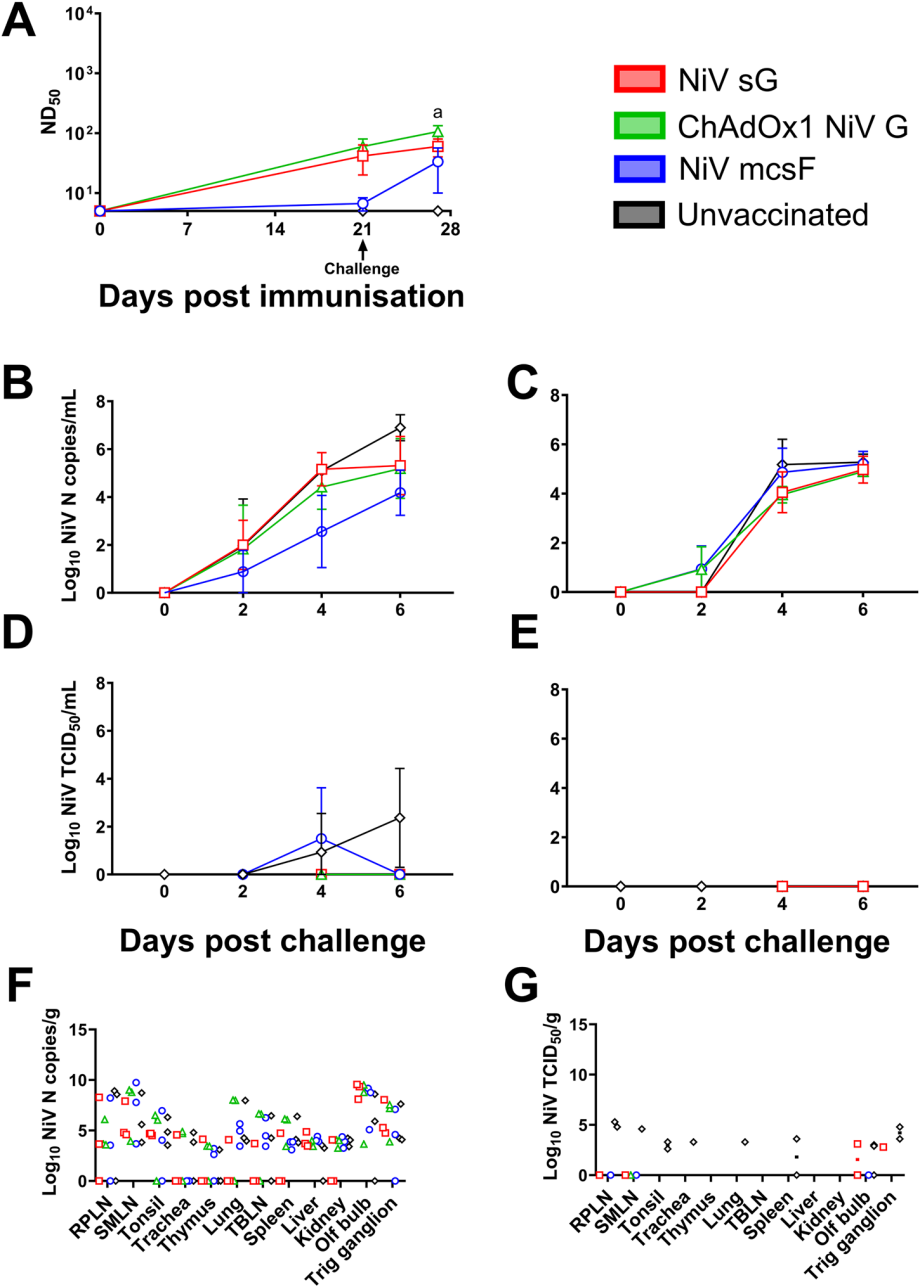
NiV vaccine candidates are not effective in pigs following a prime-only immunisation regimen. Pigs were immunised on day 0 by intramuscular inoculation of 100 µg NiV sG or NiV mcsF proteins in adjuvant, or 1 × 10^9^ IU ChAdOx1 NiV G. On day 21, all pigs were challenged by oronasal inoculation of 1 × 10^5^ PFU NiV_M_. **A** Neutralising antibody titres as assessed by NiV_M_ VNT; **B**, **C** Viral loads in nasal and oral swabs detected post-challenge by RT-qPCR, respectively. **D**, **E** Level of infectious NiV from nasal and oral swabs detected post-challenge by virus isolation, respectively. **F**, **G** Viral loads from tissue samples at 6 days post-challenge detected by RT-qPCR and virus isolation, respectively. Postmortem tissues collected: PSLN prescapular lymph node, RPLN retropharyngeal lymph node, SMLN submandibular lymph node, TBLN tracheobronchial lymph nodes, Olf bulb olfactory bulb, Trig ganglion trigeminal ganglion. Significant differences were determined using two-way ANOVA and signified with the following letter: a—significant difference to unvaccinated.

After demonstrating efficacy in pigs with all three vaccine candidates after a prime-boost regimen, efficacy after a single immunisation was determined by challenging pigs with NiV_M_ on day 21, 3 weeks post-prime (Fig. 4). Neutralising antibody titres at day 21 (Fig. 4A) were comparable to those seen in the previous immunogenicity study (Fig. 2B) and efficacy study (Fig. 3A). At 6 days post-challenge, neutralising antibody titres from pigs immunised with ChAdOx1 NiV G were significantly higher than the unvaccinated controls (*p* < 0.01), However, no significant difference in viral loads could be detected between any vaccinated groups when compared to the unvaccinated controls in either nasal (Fig. 4B) or oral swabs (Fig. 4C), or postmortem tissue samples (Fig. 4F). Infectious NiV could only be detected in nasal swabs from unvaccinated pigs on day 4 and 6 post challenge and pigs immunised with NiV mcsF on day 4 post challenge (Fig. 4D). At post-mortem, NiV RNA copies were found in all tissues sampled from unvaccinated pigs (Fig. 4F). Infectious NiV was assessed in samples which gave a CT value of ≤34. Infectious virus was isolated from tissues of pigs vaccinated with NiV sG (olfactory bulb and trigeminal ganglion) but no significance between groups was found. Infectious virus was isolated from all samples from unvaccinated controls except the thymus, liver, and kidney (Fig. 4G).

### Comparison of vaccine candidate immunogenicity following prime only and prime-boost immunisation regimens

**Fig. 5:**
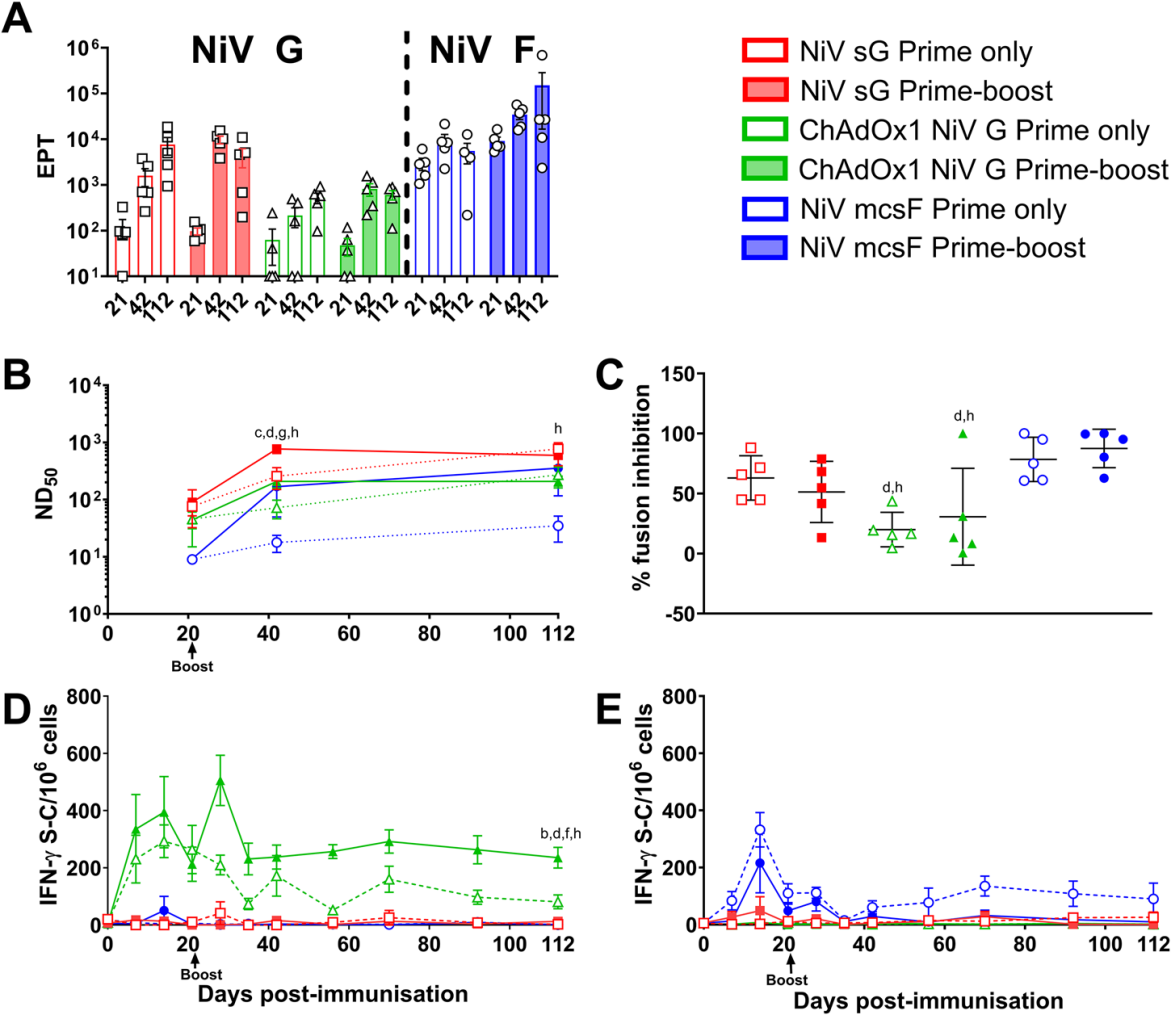
Comparison of the immunogenicity of NiV sG, ChAdOx1 NiV G, and NiV mcsF following prime only and prime-boost immunisation regimens. All pigs were immunised on day 0 and 3 out of the 6 groups were boosted on day 21 by intramuscular inoculation of 100 µg NiV sG or NiV mcsF proteins in adjuvant, or 1 × 10^9^ IU ChAdOx1 NiV G. **A** NiV sG and mcsF binding antibody titres (EPT) on day 21, 42 and 112; **B** Virus neutralising titres as assessed by NiV_M_ VNT; **C** Inhibition of NiV glycoprotein-mediated cell-cell fusion evaluated using day 112 sera; **D**, **E** IFN-γ ELISpot assay to assess PBMC responses to NiV G and F peptide stimulation, respectively. For **A** and **C**, datapoints represent individual pigs, with the bars showing the group mean and error bars represent the SD. For the other panels, datapoints represent the group mean and error bars represent the SD. Significant differences were determined using two-way ANOVA and signified with the following letter: b—significant difference to NiV sG prime-boost; c—significant difference to ChAdOx1 NiV G prime-boost; d—significant difference to NiV mcsF prime-boost; f—significant difference to NiV sG prime only; g—significant difference to ChAdOX1 NiV G prime only; h—significant difference to NiV mcsF prime only.

After showing a reduced efficacy from the vaccine candidates 21 days after a single immunisation, the three vaccine candidates were evaluated in pigs in a second immunogenicity study to determine immunological differences between the prime-only and prime-boost regimen over a 4-month time period, which is comparable to the time required to rear a weaned pig to slaughter weight (Fig. 5). No significant differences were detected in NiV G or F specific antibody titres between pigs receiving a single shot or prime boost regimen of either NiV sG, NiV mcsF or ChAdOx1 NiV G at day 21, 42 or 112 (Fig. 5A). Longitudinal NiV G and F binding antibody data is shown in Supplementary Fig. 6. However, at 112 dpv, significantly higher (*p* < 0.05) neutralising antibody titres were seen in pigs receiving a prime-only regimen of NiV sG compared to a prime-boost regimen of ChAdOx1 NiV G (Fig. 5B). Pigs receiving a prime-only regimen of NiV sG also had significantly higher (*p* < 0.01) neutralising antibody titter compared to pigs receiving a single dose of NiV mcsF (Fig. 5B). A similar trend was seen in neutralising titres using the pseudoVNT, but no significant differences were detected (Supplementary Fig. 7). All groups showed an upward trend or sustained level in neutralising titres throughout the study period. It is of interest that by day 112, pigs receiving a single immunisation with NiV sG or ChAdOx1 NiV G had similar titres to their matching vaccine group receiving a prime-boost regimen (Fig. 5B). However, there was a clear effect of the booster immunisation with NiV mcsF to augment neutralising antibody titres. When determining day 112 serum inhibition of cell-to-cell fusion, again there was no significant difference between pigs receiving the prime-only compared to prime-boost regimens in any of the vaccine groups (Fig. 5C). However, pigs receiving ChAdOx1 NiV G had significantly less ability to inhibit fusion when compared to pigs receiving NiV mcsF under both regimens (*p* < 0.05 and <0.01). Pigs immunised with ChAdOx1 NiV G again showed the highest level of IFN-γ producing cells which appeared sustained. Pigs receiving the prime-boost regimen had the highest levels of IFN-γ producing cells; significantly greater than other groups (*p* < 0.01) apart from the prime-only ChAdOx1 NiV G group (Fig. 5D). NiV mcsF prime-only vaccinated pigs had the highest, and sustained, F specific T-cell responses, which at 112 dpv was significantly (*p* < 0.0001) higher than all other groups (Fig. 5E). As seen in the first pig immunogenicity study (Supplementary Figs. 4 and 5), pigs receiving NiV sG displayed spontaneous T-cell IFN-γ responses (Supplementary Fig. 8).

### Evaluation of vaccine candidate immunogenicity in pigs under field conditions in the Nipah virus endemic region

**Fig. 6:**
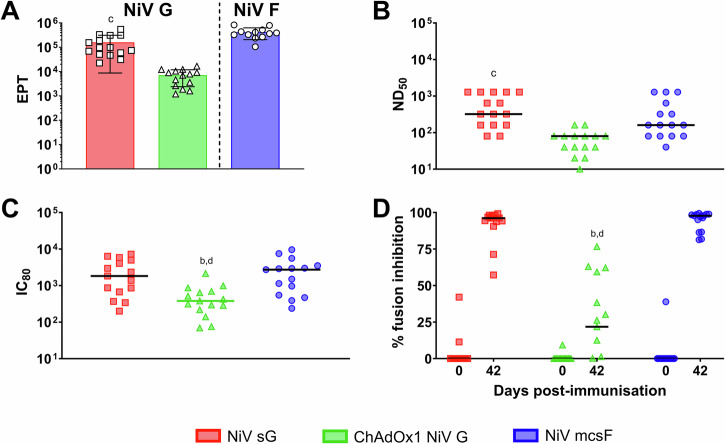
Immunogenicity of NiV sG, ChAdOx1 NiV G and NiV mcsF in indigenous backyard pigs under field conditions in the ‘Nipah belt’ of Bangladesh. Pigs were immunised on day 0 and 21 by intramuscular inoculation of 100 µg NiV sG or NiV mcsF proteins in adjuvant, or 1 × 10^9^ IU ChAdOx1 NiV G. **A** NiV mcsF and sG binding antibody titres (EPT) on day 42; **B**, **C** Virus neutralising titres as assessed by NiV_M_ VNT and pseudoVNT, respectively; **D** Inhibition of NiV glycoprotein-mediated cell-cell fusion evaluated using day 42 sera. Significant differences were determined using one-way ANOVA (**A**, **B** and **D**) or two-way ANOVA (**C**) and signified with the following letter: b—significant difference to NiV sG; c—significant difference to ChAdOx1 NiV G; d—significant difference to NiV mcsF.

A final assessment of the vaccine candidates was undertaken in a NiV endemic region of Bangladesh, immunising indigenous breed pigs under field conditions (Fig. 6). An initial pre-screen for antibody reactivity to NiV sG and mcsF was undertaken using sera from 60 pigs (Supplementary Fig. 9). Five pigs with an OD value higher than twice the negative control were excluded from the study. Immunisation with the three vaccine candidates produced NiV G or F specific antibody titres (Fig. 6A), comparable to those elicited in the European breed pigs under controlled conditions (Figs. 2A and 5A). The indigenous backyard pigs immunised with NiV mcsF produced higher neutralising antibody titres (Fig. 6B) compared to the previous studies (Figs. 2B, 3A, 4A and 5B) with pigs immunised with ChAdOx1 NiV G producing a reduced neutralising titter compared to NiV sG immunised pigs (*p* < 0.01). These data were also replicated in the pseudoVNT assay (Fig. 6C). When determining the ability of vaccinated pig sera to inhibit cell-to-cell fusion, at 42 dpv, pigs receiving the ChAdOx1 NiV G had a significantly reduced ability to do so compared to pigs immunised with NiV sG and NiV mcsF (*p* < 0.0001) (Fig. 6D).

## Discussion

The ‘One Health’ strategy aligns veterinary and medical public health efforts, ensuring comprehensive surveillance and response measures. This integrative approach not only addresses immediate infection control but also supports long-term prevention and preparedness against emerging infectious diseases^11^. The vaccination of pigs, as primary amplifying hosts of NiV, represents a strategic intervention that could significantly reduce the risk of zoonotic transmission to humans^25^. By preventing NiV outbreaks in swine populations, we can in turn mitigate human infections, protect economies, enhance public health security, and provide food security.

**Fig. 7:**
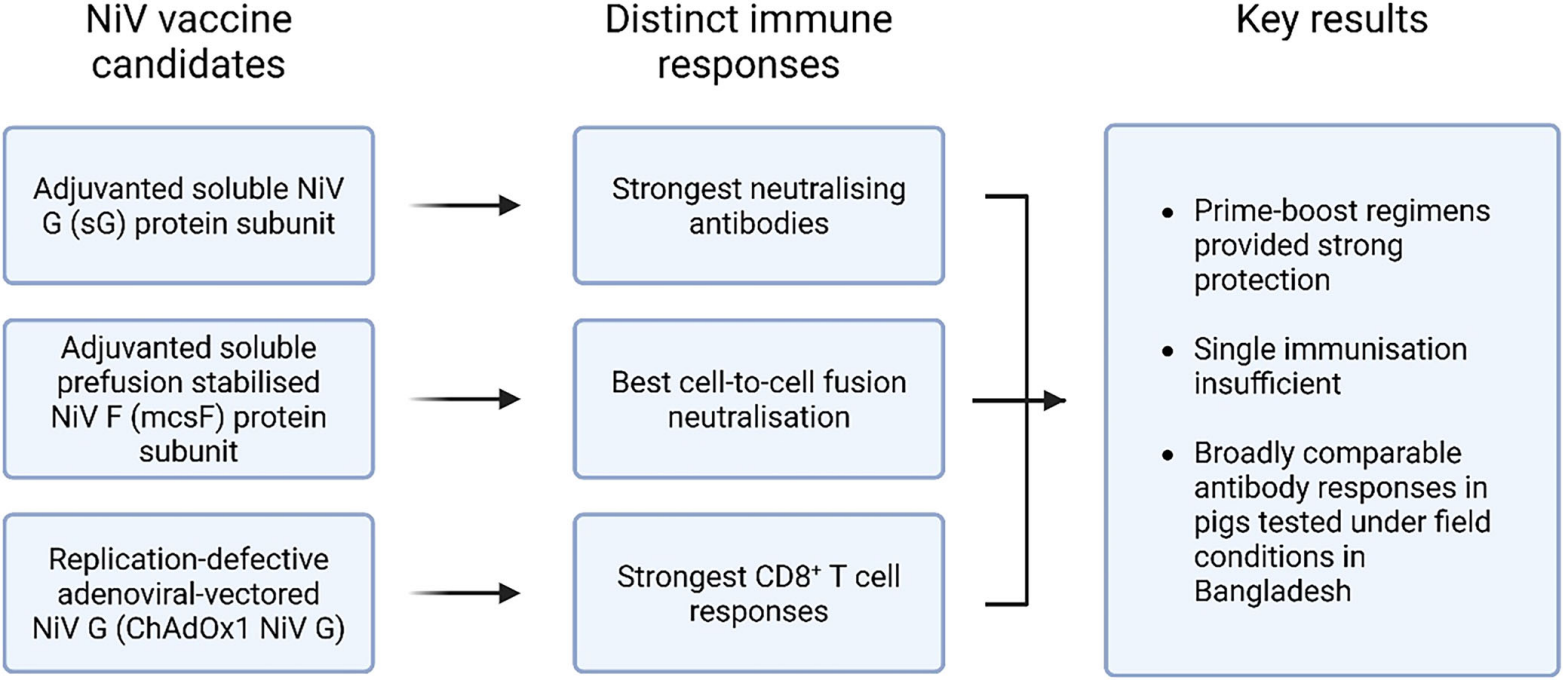
Schematic summary of the evaluation of NiV vaccine candidates in pigs. Created in BioRender.

This study demonstrated that immunisation of pigs with NiV sG, ChAdOx1 NiV G or NiV mcsF in a prime-boost regimen significantly reduced viral shedding and tissue loads following NiV challenge infection. The highest levels of NiV neutralising antibodies were induced in both mice and pigs immunised with NiV sG, whereas those immunised with ChAdOx1 NiV G elicited the only significant CD8^+^ T-cell response. Pigs immunised with NiV mcsF showed the greatest ability to inhibit cell-to-cell fusion. It has previously been hypothesised for SARS-CoV-2 and respiratory syncytial virus (RSV) that the development of a highly fusion-inhibitory antibody response may correlate well with protection from infection^26^ which could also be associated to protection against NiV. Despite these differences in immune responses, all three vaccine candidates similarly protected pigs (Fig. 7). As there was no detectable infectious virus in oral or nasal swabs or tissues from vaccinated pigs, the data suggest that the vaccines would limit or prevent the onward transmission of NiV to uninfected animals or humans. Whilst challenging under BSL-4 containment, future studies should also evaluate the prevention of transmission by the inclusion of sentinel pigs during the post-challenge period.

The virus dose used for challenge was based on previous efficacy studies in pigs^15,27,28^ and additional data from ACDP, Australia and the National Centre for Foreign Animal Disease, Canada (not cited). To avoid severe clinical disease but have a confirmed infection, a dose of 1 × 10^5^ PFU of NiV_M_ was used. At this dosing regimen, infectious virus is still shed from pigs, and it infects multiple organs, including the nervous system of the pig. Experimental infection of pigs with NiV_M_ and NiV_B_ is an acute and self-limiting infection, with detectable shedding of NiV-M and NiV-B up to 8–9 days post-infection^15,27–29^. Therefore, the efficacy study design utilised here was intended to determine protection by reduction in viral shedding and viral loads in tissues collected post-mortem 6 days post-challenge, at the peak of infection. However, future studies should additionally assess protective efficacy over a longer period post-challenge to offer more insight into the kinetics of protection and the potential for viral persistence or rebound.

Given the acute nature of NiV infections in humans and pig production practices, a vaccine that is effective after a single immunisation is more desirable. Pigs are typically reared in large populations where rapid transmission of pathogens can occur, requiring swift and robust immunity to prevent outbreaks. Moreover, the logistical challenges and costs associated with multiple vaccinations in large-scale pig farming make single-dose vaccines economically and practically advantageous. Hence, for a NiV vaccine to be adopted successfully in swine populations in at-risk countries, it should ideally confer long-lasting immunity after a single immunisation. This approach not only ensures a more rapid onset of immunity but also minimises labour and costs. A single immunisation with NiV sG, ChAdOx1 NiV G or NiV mcsF was insufficient to provide effective protection against NiV, at least when pigs were challenged after 21 days. However, the subsequent immunogenicity study showed that antibody titres continued to rise following 21 days, which suggest that it may be worthwhile testing if protective immunity could be provided by a single dose at a later timepoint. Ensuring a long-term immune response against NiV through vaccination in pigs is crucial for several reasons. Long-lasting immunity reduces the need for frequent re-vaccination, thereby lowering operational costs and labour in large-scale pig farming. Moreover, sustained immunity in pigs is essential to reduce the risk of outbreaks with substantial economic losses and risk to public health. Therefore, future studies should determine the duration of immunity provided by the NiV vaccine candidates and explore the use of increased doses and additional antigens to achieving long-lasting protection by a single immunisation with these NiV vaccine candidates.

Despite the risk NiV poses, developing a commercial NiV vaccine for pigs is challenging. The rare and sporadic nature of NiV spillover into pigs means that there will be limited marketability. Two scenarios may be envisaged for how a NiV vaccine for pigs could be deployed: (1) as part of a government vaccine bank and used in an outbreak situation; or (2) incorporated into routine vaccination programs to reduce the risk of NiV outbreaks occurring. For the former, a vaccine that provides a rapid onset of immunity after a single immunisation would be preferable, and for the latter, a dual purpose/bivalent vaccine would be a more financially viable approach^30^. The data have shown both NiV F and G to be protective antigens in pigs. Building on this, we are now evaluating the potential for live attenuated pseudorabies virus expressing both NiV glycoproteins to satisfy both scenarios i.e., provide rapid immunity after a single immunisation and could also be used routinely as a bivalent vaccine in pig populations at risk from NiV. Additionally, as intramuscular immunisation is not the most effective way to stimulate mucosal responses, the route of vaccine delivery should be investigated in a future study. Considering the natural transmission route of NiV in pigs is via respiratory droplets, oral and nasal delivery of these vaccine candidates should be assessed. Previous success has been shown with oronasal delivery over intramuscular against influenza A virus where potent mucosal responses were detected^31^.

Furthermore, since the pig provides a useful preclinical biomedical model^19^, the immunogenicity and efficacy of the vaccine candidates detailed in this study are relevant to NiV vaccine development for humans, and supported ChAdOx1 expressing NiV_B_ G entering a phase I clinical trial^32^. Other studies have demonstrated that vaccines tested in pigs often yield predictive data relevant to human health outcomes, thereby enhancing the reliability of preclinical vaccine assessments^24,33^. Consequently, incorporating pigs into vaccine research can bridge the gap between animal models and human clinical trials, accelerating the development of effective vaccines for human use. As an example, during the COVID-19 pandemic, we first assessed the immunogenicity of potential vaccines against SARS-CoV-2 in pigs to predict the responses in humans^24^.

In conclusion, this study has demonstrated the immunogenicity of NiV sG, ChAdOx1 NiV G and NiV mcsF vaccine candidates in pigs, including in backyard indigenous breed pigs reared in areas where NiV outbreaks occur. Despite inducing a distinct profile of immune responses, all three vaccines significantly reduced NiV shedding and loads in pigs, when used in a prime-boost regimen. The protection conferred would reduce, if not prevent, NiV transmission which would safeguard human health and reduce the socioeconomic impact associated with outbreaks. However, the feasibility of commercially developing and deploying a NiV vaccine for pigs remains to be determined and alternative mechanisms may provide a more sustainable alternative.

## Methods

### Ethics statement

Animal studies were performed in accordance with the relevant national legislation (UK—Animals (Scientific Procedures) Act 1986, Australia—Australian Code for the Care and Use of Animals for Scientific Purposes (8th Edition), and Bangladesh (Research Review Committee (RRC) and Animal Experimentation Ethical Committee (AEEC) of icddr,b; and the Department of Livestock Services (DLS) and Directorate of Drug Administration, Government of the People’s Republic of Bangladesh) and approved by the relevant local Animal Welfare and Ethical Review Bodies (AWERB) (UK: Mice—Project License 30/2889 and University of Oxford AWERB, and pigs—Project License P9C86DC55 and The Pirbright Institute and Animal and Plant Health Agency (APHA) AWERBs; Australia: CSIRO ACDP Animal Ethics Committee (AEC approval #1948); and Bangladesh: Protocol PR-19068, icddr,b Research Review and Animal Experimentation Ethical Committees). The principles of the 3R’s were applied for the duration of the study to ensure animal welfare was not unnecessarily compromised.

### Vaccines

The construction and preparation of ChAdOx1 expressing NiV_M_ G protein (GenBank Accession number NP_112027.1) (ChAdOx1 NiV G) was as described previously for ChAdOx1 NiV_B_ G protein^20^. Recombinant soluble NiV_M_ G (NiV sG) (GenBank Accession number NP_112027.1) and molecular clamp stabilised NiV_M_ F protein (GenBank Accession number NP_112026.1) (NiV mcsF) proteins were expressed and purified as described previously^34^. Recombinant soluble Hendra G protein (HeV sG), the antigen used in the Equivac® HeV vaccine for horses, Zoetis Australia Pty Ltd., was produced in CHO cells. Recombinant proteins and ChAdOx1 NiV G were filter sterilised using a 0.22 μm syringe filter and a confirmed to contain <5 EU/mL endotoxin using the Pierce™ Chromogenic Endotoxin Quant Kit (Thermo Fisher Scientific). NiV sG, NiV mcsF and HeV sG proteins were formulated in a ZAF-001, proprietary oleaginous based water in oil emulsion containing surfactants, ionic polymer and a synthetic immunomodulator (Zoetis Inc, Kalamazoo, MI, USA).

### Vaccine trials

#### Vaccine immunogenicity in mice

Female BALB/cOlaHsd (BALB/c) mice (Envigo) were randomly allocated to five treatment groups upon arrival. At 9–10 weeks of age, groups of 9–10 mice were immunised intramuscularly (inoculum volume 50 μL) with 1 × 10^8^ infectious units (IU) (1 × 10^10^ virus particles; vp) ChAdOx1 NiV G; 5 μg NiV sG in ZAF-001; 5 μg NiV mcsF in ZAF-001; 5 μg HeV sG in ZAF-001; or an equivalent volume (50 μL) of ZAF-001. After 21 days, 4–5 mice/group were euthanised to enable collection of serum and spleens, and the remaining mice were given a booster immunisation. Spleens and serum were harvested from the boosted animals a further 3 weeks later. All mice were humanely sacrificed by cervical dislocation.

#### Prime-boost vaccine immunogenicity in pigs

Forty-five, 8–10-week-old, female, Large White-Landrace-Hampshire cross-bred pigs were randomly assigned to the same five treatment groups. Pigs were immunised by intramuscular inoculation (inoculum volume 1 mL) of either 1 × 10^9^ IU (1 × 10^11^ vp) ChAdOx1 NiV G, 100 μg NiV sG in ZAF-001; 100 μg NiV mcsF in ZAF-001; 100 μg HeV sG in ZAF-001; or an equivalent volume (1 mL) of ZAF-001. After 3 weeks, 3 animals per group were euthanised. Animals were sedated by intramuscular injection with a cocktail of Domitor (Medetomidine; 1 mg/mL) and Zoletil (Tiletamine and Zolazepam; 50 mg/mL) at a concentration of 0.5 mL/10 kg body weight, before an overdose of pentobarbital sodium anaesthetic (Pentoject—20 mL/animal) by intravenous injection in the marginal ear vein, followed by exsanguination, to enable post-mortem collection of tissue samples. The remaining 6 animals per group received a homologous booster immunisation and were euthanised as described above after a further 3 weeks. Animals were scored daily for clinical signs and rectal temperature measurements (Supplementary Table 1), and blood samples were collected weekly.

#### Prime only versus prime-boost vaccine immunogenicity in pigs

Thirty, 8–10-week-old, female, Large White-Landrace-Hampshire cross-bred pigs were randomly assigned to six treatment groups. Groups of 5 pigs were immunised once or twice (21-day interval) by intramuscular injection (inoculum volume 1 mL) with 1 × 10^9^ IU (1 × 10^11^ vp) ChAdOx1 NiV G, 100 μg NiV sG in ZAF-001; or 100 μg NiV mcsF in ZAF-001. Blood samples were collected on 0, 7, 14, 21, 28, 35, 42, 56, 70, 91 and 112 days post-vaccination (dpv). All animals were then euthanised as described above.

#### Immunogenicity in pigs under field conditions

Serum samples were collected from 60 mixed sex indigenous Deshi breed pigs (3–12 months of age) reared under ‘backyard’ production systems in the Godagari sub-district of Rajshahi district in Bangladesh. Sera were screened by ELISA to assess reactivity against NiV sG and mcsF proteins by ELISA^34^. Pigs which showed reactivity to either antigen (threshold for reactivity defined as twice the mean optical density (OD) 450 nm (OD_450_) for NiV naïve sera) were excluded (Supplementary Fig. 9). Forty-five ELISA negative pigs distributed in three separate geographical clusters for the convenience of the study (Cluster A—one village (Taltipara); Cluster B—one village (Adar-para); and Cluster C—five villages (Nandapur, Bilashi, Shah-para Jhina Fulbari and Proshad-para) (15 pigs/cluster) were enrolled in the study based on feedback from the field team. Pigs in each cluster were randomly assigned to three treatment groups (*n* = 15) and immunised on days 0 and 21 by intramuscular inoculation (inoculum volume 1 mL) with 1 × 10^9^ IU (1 × 10^11^ vp) ChAdOx1 NiV G, 100 μg NiV sG in ZAF-001; or 100 μg NiV mcsF in ZAF-001. Pigs were clinically monitored for 7 days after each immunisation and blood sampled on 7, 14, 28 and 42 dpv prior to euthanasia. Pigs were first sedated by intramuscular injection with Easium®, followed by inoculation of thiopental sodium (TPS®, Popular Pharmaceuticals Limited, Bangladesh) intravenously into the anastomosis of the external and internal jugular vein. The heart rate was continuously monitored using a stethoscope by a veterinarian. The death of a pig was declared by the veterinarian when there no heartbeat was heard. Absence of a corneal reflex and absence of breathing was also observed before death pronounced.

#### Assessment of vaccine efficacy in pigs

A vaccine efficacy study was run as three experimental replicates. In each replicate, four female, commercial Large White/Landrace/Duroc cross, weanling (4–5 weeks of age; younger pigs were used in efficacy studies as these smaller animals were easier to handle in BSL-4 biocontainment during and following NiV challenge) pigs were randomly assigned to one of four groups: (1) unvaccinated challenge control; (2) NiV sG in ZAF-001 vaccine group; (3) NiV mcsF in ZAF-001 vaccine group; and (4) ChAdOx1 NiV G vaccine group. Pigs were vaccinated on 0 and 21 dpv as described above. On 42 dpv, all pigs were challenged by oronasal inoculation (1 mL per nostril, 1 mL orally) with 1 × 10^5^ plaque forming units (PFU) of NiV_M_. The NiV_M_ was isolated from the cerebrospinal fluid of a patient who died from encephalitis in Malaysia in 1999, and the stock utilised for these studies was a P4 stock passaged in Vero cells^27^. This NiV_M_ stock was fully sequenced (99.98% match to GenBank Accession number 002728), negative for mycoplasma, and negative for contamination with other viruses or bacteria, as assessed by next-generation sequencing. Animals were blood sampled on 0, 21, 42, 44, 46, and 48 dpv to enable assessment of immune responses (0, 21, 42, and 48 dpv) and NiV viremia (42, 44, 46, and 48 dpv). Oral, nasal, and rectal swabs were collected on 42, 44, 46, and 48 dpv to assess the shedding of NiV. Animals were euthanised on 48 dpv (6 days post-challenge) to allow the assessment of viral loads in tissues. Pigs were anaesthetised by intramuscular injection with tiletamine/zolazepam (2–4 mg/kg) and xylazine (1–2 mg/kg). Following collection of terminal bleeds from anaesthetised animals, they were euthanised by administration of up to 150 mg/kg pentobarbitone intravenously. A second efficacy trial was conducted as described above with the exception that pigs were challenged with NiV_M_ 21 days after a single immunisation. One pig in each of the NiV mcsF and control groups were found dead due to adverse reactions following anaesthesia, unrelated to vaccination or challenge.

### Assessment of vaccine immunogenicity

#### Mice

Serum was isolated from blood samples collected by cardiac bleed. Detection of NiV sG and NiV mcsF specific antibody titres were determined by ELISA^34^. Serial dilutions of 21 and 42 dpv serum samples were evaluated and endpoint titres (EPT) calculated as the reciprocal of the highest dilution at which the OD value was greater than the cut-off value determined as twice the mean OD for sera from the adjuvant only group. Detection of NiV_M_ neutralising antibodies in sera was performed by virus neutralisation test (VNT) as previously described^35^. Neutralisation titres were expressed as the reciprocal of the serum dilution that completely blocked cytopathic effect in 50% of wells (ND_50_). Single cell suspensions of splenocytes were stimulated with pools of overlapping synthetic peptides (16mers offset by 4 amino acids) representing NiV_M_ G and F proteins (Mimotopes, Melbourne, Australia) and responses assessed by flow cytometric intracellular cytokine assays as described previously^24^. Splenocytes from all groups were restimulated with NiV_M_ G peptides, whereas splenocytes from the NiV sG, NiV mcsF and adjuvant only groups were restimulated with NiV_M_ F peptides.

#### Pigs

Serum and peripheral blood mononuclear cells (PBMC) were isolated from blood collected in SST and heparin vacutainers (Thermo Fisher Scientific), respectively^34^. PBMC responses to stimulation with NiV_M_ G and F peptide pools were assessed by IFN-γ ELISpot and flow cytometric intracellular cytokine assays as described previously^34^. Detection of NiV F- and G-specific antibodies was performed in sera^34^; all serum samples were first assessed in a IgG-specific indirect ELISA at a 1:400 dilution and end point titres were determined for selected timepoint samples as described above. Quantification of NiV_M_ neutralising antibodies in sera was performed by VNT as described above. Detection of neutralising antibodies was also conducted in serum samples using NiV_M_ pseudovirus (pseudoVNT) as described^26,34^. Pseudovirus neutralisation titres were calculated as the inverse of the dilution which showed an 80% inhibition of luciferase values (IC_80_), compared to no serum controls. Selected sera were also assessed for neutralisation of NiV_M_ glycoprotein-mediated cell–cell fusion using a quantitative fusion assay (mFIT), and data calculated as the percentage of reduction of luciferase values compared to no sera control^26,34^.

### Assessment of vaccine efficacy

Nasal, oral, and rectal swabs were collected using cotton swabs (sterile rayon transport swab with wood shaft (LabCo Scientific Australia)) and placed into 1 mL transport media (MEM (Thermo Fisher Scientific) with 5× Antibiotic/Antimycotic (Thermo Fisher Scientific) and 0.1% BSA (Merck)). Plasma samples were prepared from EDTA blood samples. Tissue samples (prescapular, retropharyngeal, tracheobronchial, and submandibular lymph nodes, tonsils, trachea, thymus, lung, spleen, liver, kidney, olfactory bulb, trigeminal ganglion, and nasal turbinates) were collected post-mortem 6 days after NiV challenge. RNA was extracted from samples using the MagMax Viral RNA isolation kit (Thermo Fisher Scientific) following the manufacturer’s recommendations and NiV load inferred by RT-qPCR^36^. Infectious NiV titres in samples with Cq values <34 were determined by virus isolation assay on Vero cells^36^.

### Data analysis

GraphPad Prism 10.1.2 (GraphPad Software, San Diego, CA, USA) was used for graphical and statistical analysis of data sets. T-cell data are presented as the unstimulated condition-corrected number/frequency of cytokines producing cells. Statistical differences were analysed using either one-way or two-way ANOVA, or a mixed-effects model followed by a Šidák´s multiple comparison test to compare antigen-specific cytokine and antibody responses at different time points post-vaccination as detailed in the results. Antibody titre data were log_10_ transformed before analysis. *p* values < 0.05 were considered statistically significant.

## Supplementary information

Supplementary Information

## Data Availability

The data that support the findings of this study are available from the corresponding author upon request.
