# Supplementary material for: Nipah virus vaccines evaluated in pigs as a ‘One Health’ approach to protect public health
Source: NPJ Vaccines. 2025 Jul 23;10:163. doi: 10.1038/s41541-025-01212-y (PMC12287429; doi:10.1038/s41541-025-01212-y)
Supplement: Supplementary file 1 — Supplementary Information [file 41541_2025_1212_MOESM1_ESM.pdf]

McLean et al., Nipah virus vaccines evaluated in pigs as a 'One Health' approach to protect public health

Supplementary Material

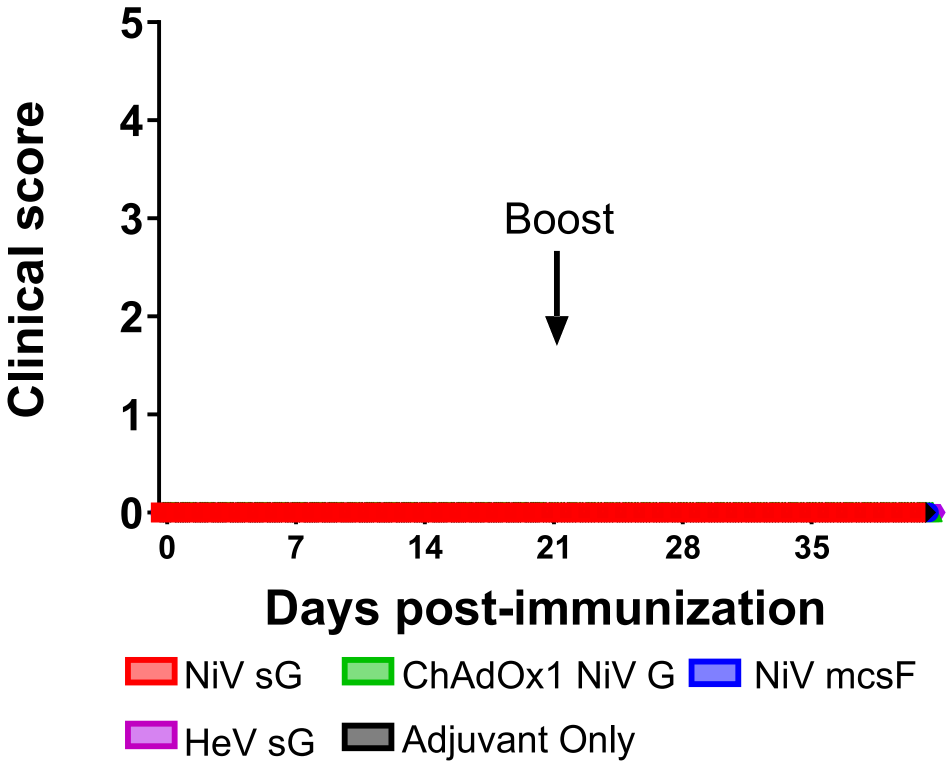

Supplementary Figure 1: Evaluation of the reactogenicity of NiV vaccine candidates in pigs. Pigs were immunized on day 0 and 21 by intramuscular inoculation of 100 µg NiV sG, NiV mcsF, or HeV sG proteins in adjuvant, or 1 × 10^9 IU ChAdOx1 NiV G. Rectal temperatures were measured and combined with other clinical signs to create a clinical score (**Supplementary Table 1**) daily until 41 days post-vaccination. Mean score value ± SD for each group are shown.

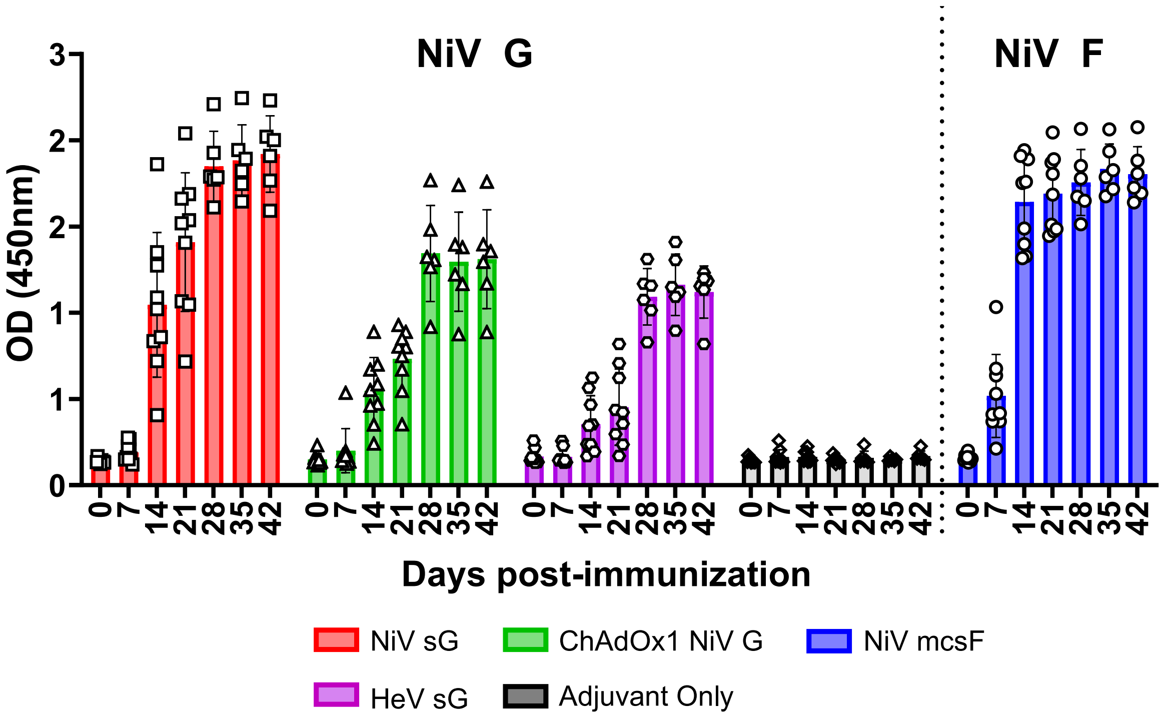

Supplementary Figure 2: Antibody responses of pigs following immunization with NiV vaccine candidates. Pigs were immunized on day 0 and 21 by intramuscular inoculation of 100 µg NiV sG, NiV mcsF, or HeV sG proteins in adjuvant, or 1 × 10^9 IU ChAdOx1 NiV G. NiV sG or mcsF binding antibodies were determined in weekly collected sera by ELISA. Results are shown as optical density (OD; 450nm) measurements. Datapoints represent individual pigs, with the bars showing the group mean and error bars representing the SD.

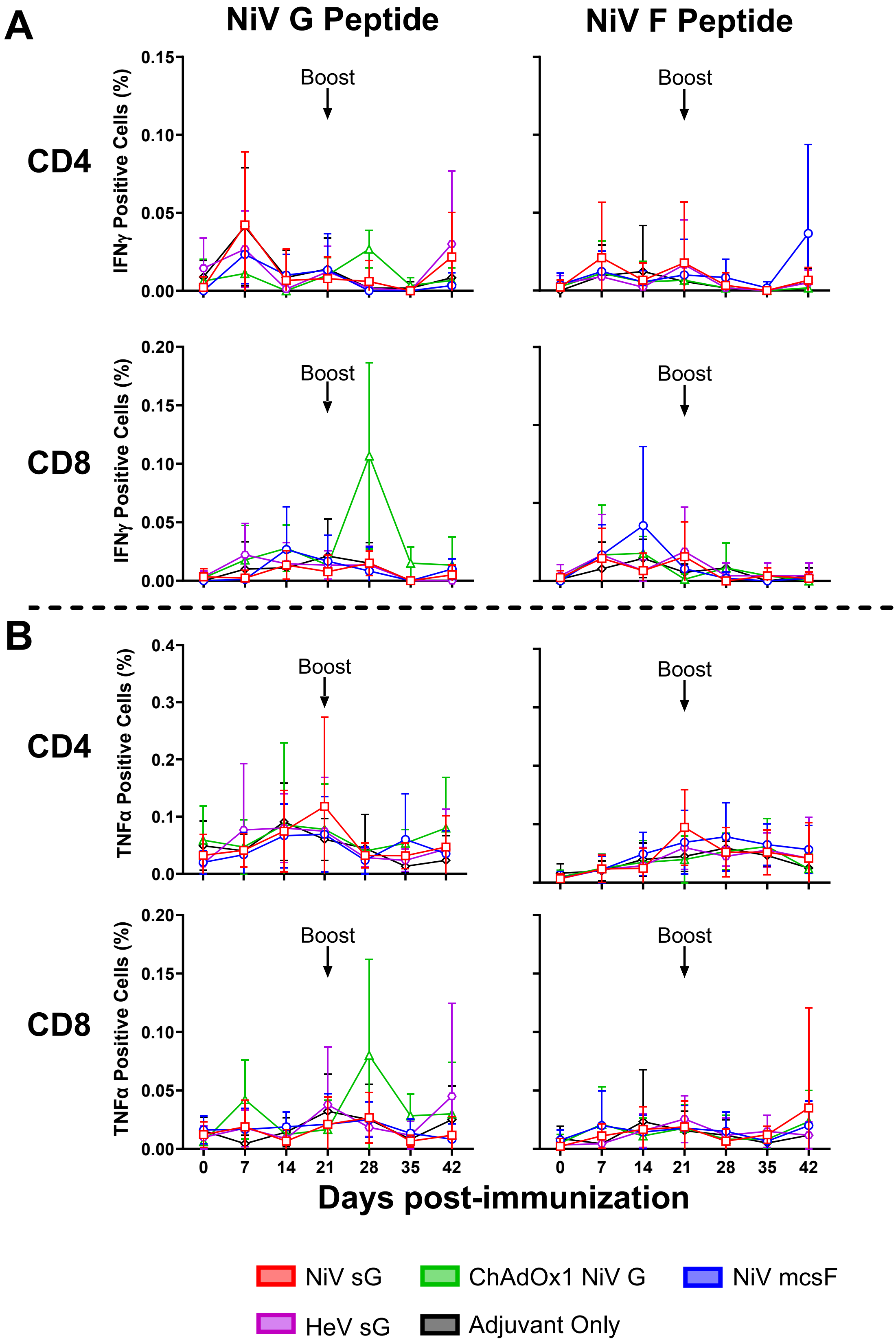

Supplementary Figure 3: Assessment of CD4$^+$ and CD8$^+$ T-cells producing only (**A**) IFN-γ or (**B**) only TNF-α after stimulation with NiV G or F peptides. Pigs were immunized on day 0 and 21 by intramuscular inoculation of 100 µg NiV sG, NiV mcsF, or HeV sG proteins in adjuvant, or 1 × 10^9 IU ChAdOx1 NiV G. PBMC isolated at 0, 7, 14, 21, 28, 35 and 42 dpv were stimulated with peptide pools, and analyzed by flow cytometry. Datapoints represent the group mean and error bars represent the SD.

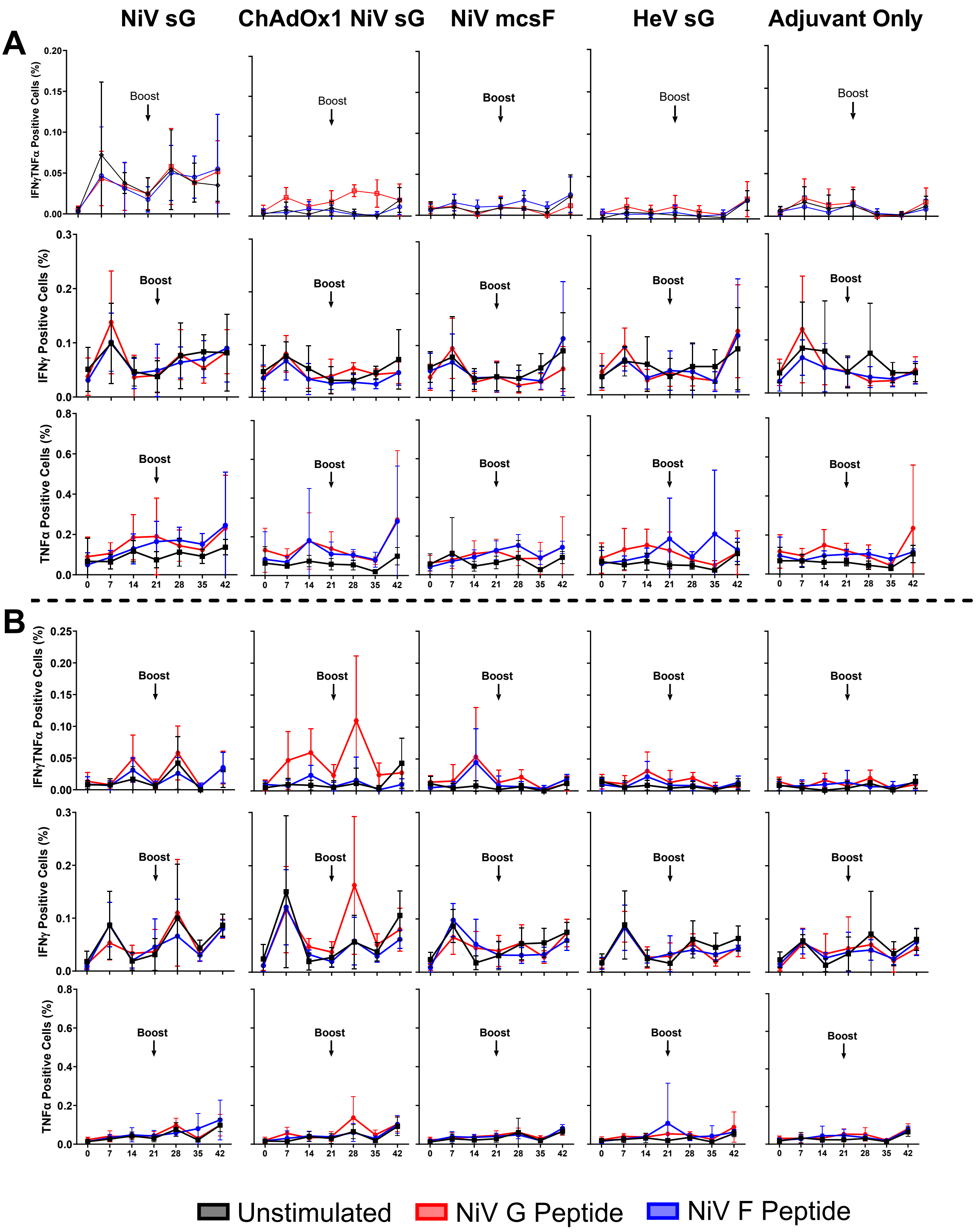

Supplementary Figure 4: Uncorrected ICS data showing non-specific T-cell IFN-γ responses from NiV sG immunized pigs. Pigs were immunized on day 0 and 21 by intramuscular inoculation of 100 µg NiV sG, NiV mcsF, or HeV sG proteins in adjuvant, or 1 × 10^9 IU ChAdOx1 NiV G. PBMC isolated at 0, 7, 14, 21, 28, 35 and 42 dpv were unstimulated or stimulated with NiV G and F peptides and labelled for ICS to assess IFN-γ and/or TNF-α expression by (**A**) $CD4^+$ and (**B**) $CD8^+$ T-cells. Datapoints represent the group mean and error bars represent the SD.

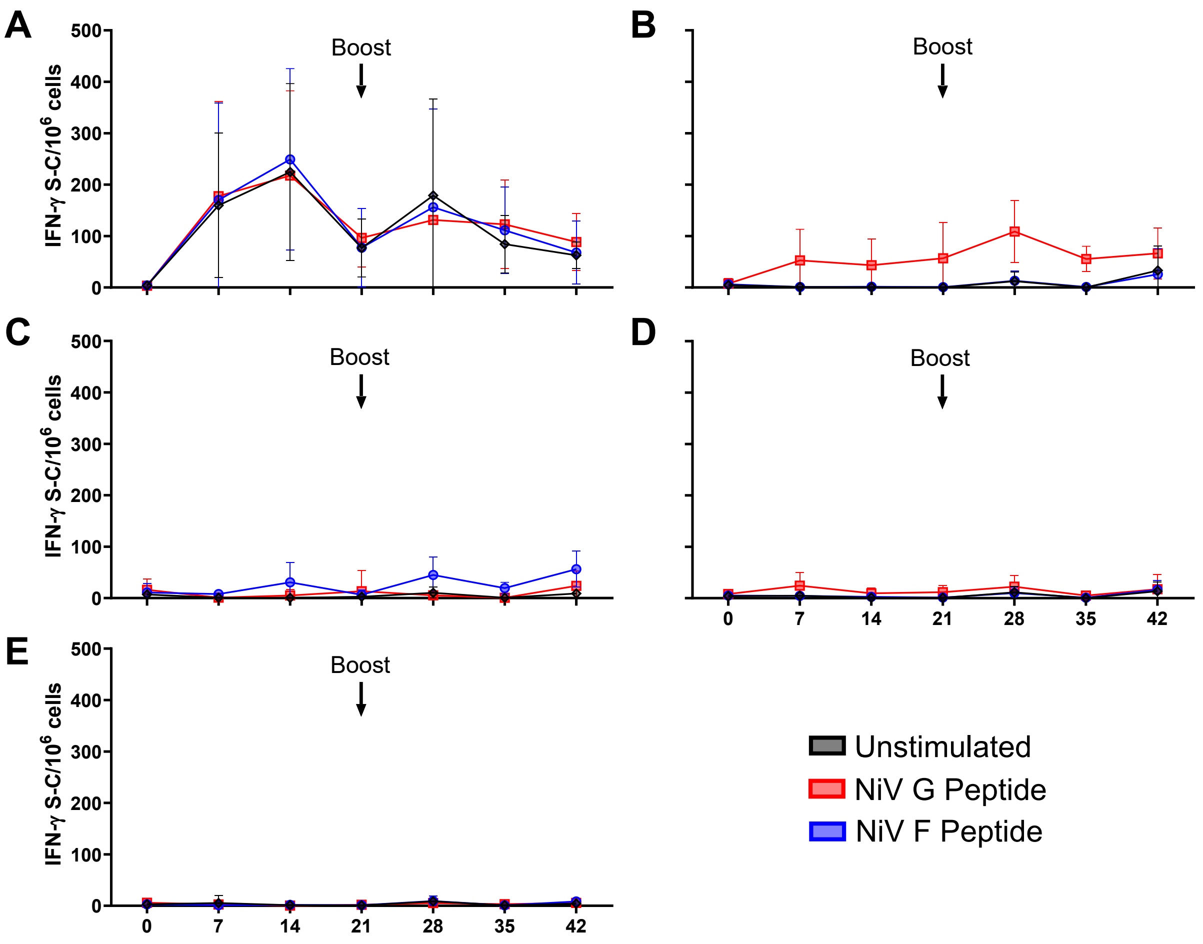

Supplementary Figure 5: Uncorrected ELISpot assay data showing non-specific T-cell IFN-γ responses from NiV sG immunized pigs. Pigs were immunized on day 0 and 21 by intramuscular inoculation of (**A**) 100 µg NiV sG, (**B**) 1 × 10^9 IU ChAdOx1 NiV G, (**C**) 100 µg NiV mcsF, (**D**) 100 µg HeV sG, or (**E**) adjuvant alone. PBMC isolated at 0, 7, 14, 21, 28, 35 and 42 dpv were unstimulated or stimulated with NiV G and F peptides and IFN-γ secreting cells assessed by ELISpot assay. Datapoints represent the group mean and error bars represent the SD.

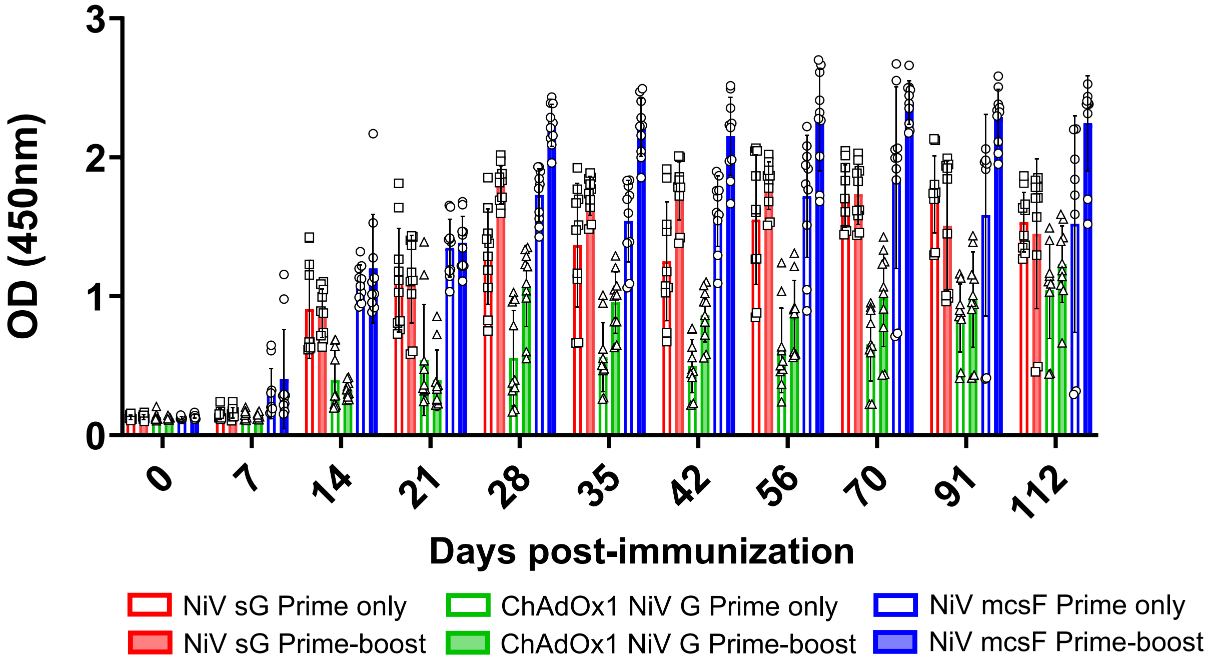

Supplementary Figure 6: Antibody responses of pigs following immunization with NiV sG, ChAdOx1 NiV G, NiV mcsF. Pigs were immunized on day 0 (prime only) or on day 0 and 21 (prime-boost) by intramuscular inoculation of 100 µg NiV sG or NiV mcsF proteins in adjuvant, or 1 × 10^9 IU ChAdOx1 NiV G. NiV sG (NiV sG and ChAdOx1 NiV G immunized groups) or mcsF (NiV mcsF immunized groups) binding antibodies were determined in sera by ELISA. Results are shown as optical density (OD; 450 nm) measurements. Datapoints represent the group mean and error bars represent the SD.

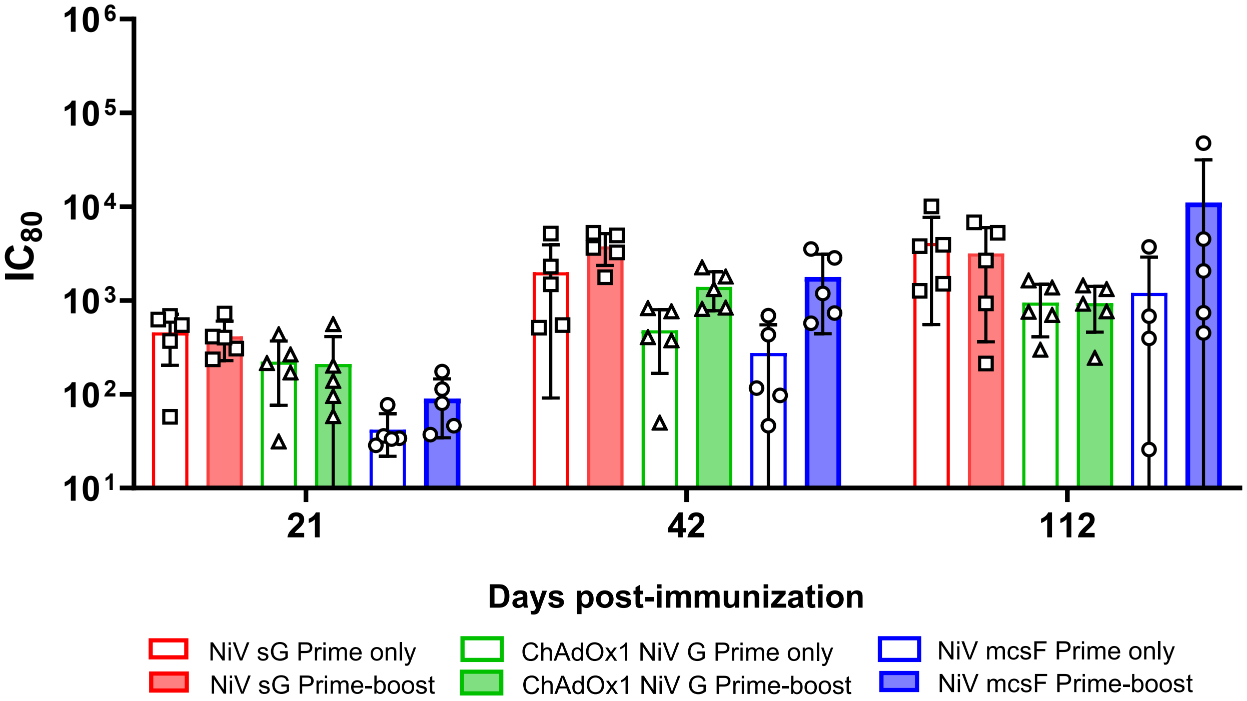

Supplementary Figure 7: Neutralizing antibody responses of pigs following immunization with NiV sG, ChAdOx1 NiV G or NiV mcsF. Pigs were immunized on day 0 (prime only) or on day 0 and 21 (prime-boost) by intramuscular inoculation of 100 µg NiV sG or NiV mcsF proteins in adjuvant, or 1 × 10^9 IU ChAdOx1 NiV G. NiV_M neutralizing antibodies were determined in days 21, 42 and 112 sera by pseudoVNT and results shown as IC_{80}. Datapoints represent the group mean and error bars represent the SD.

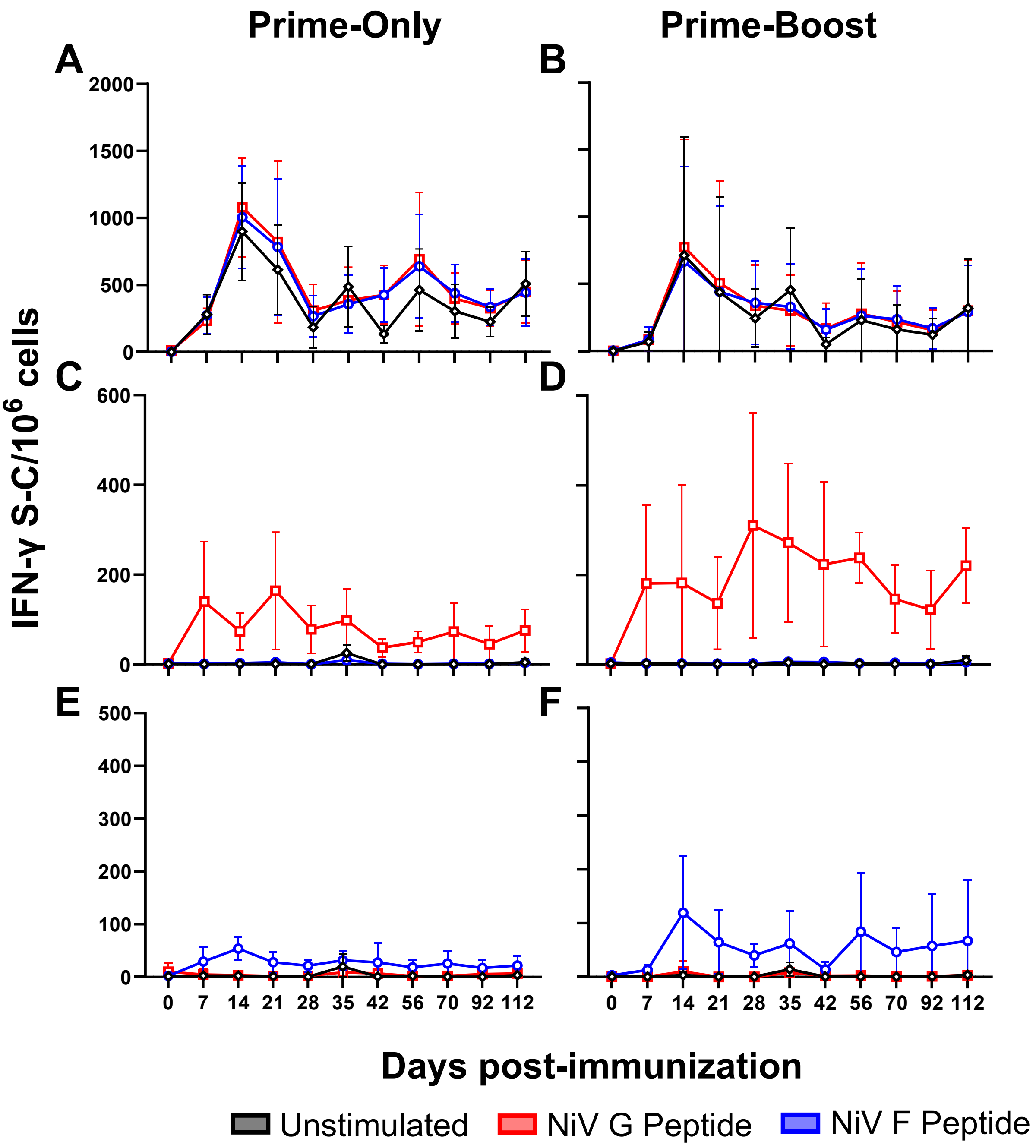

Supplementary Figure 8: Uncorrected IFN-γ ELISpot data showing non-specific responses from NiV sG immunized pigs with or without a booster immunization. Pigs were immunized on day 0 (prime only) or on day 0 and 21 (prime-boost) by intramuscular inoculation of (**A** and **B**) 100 µg NiV sG in adjuvant, (**C** and **D**) 1 × 10^9 IU ChAdOx1 NiV G, or (**E** and **F**) 100 µg NiV mcsF protein in adjuvant. PBMC isolated at 0, 7, 14, 21, 28, 35, 42, 56, 70, 92 and 112 dpv were unstimulated or stimulated with NiV G and F peptides and IFN-γ secreting cells assessed by ELISpot assay. Datapoints represent the group mean and error bars represent the SD.

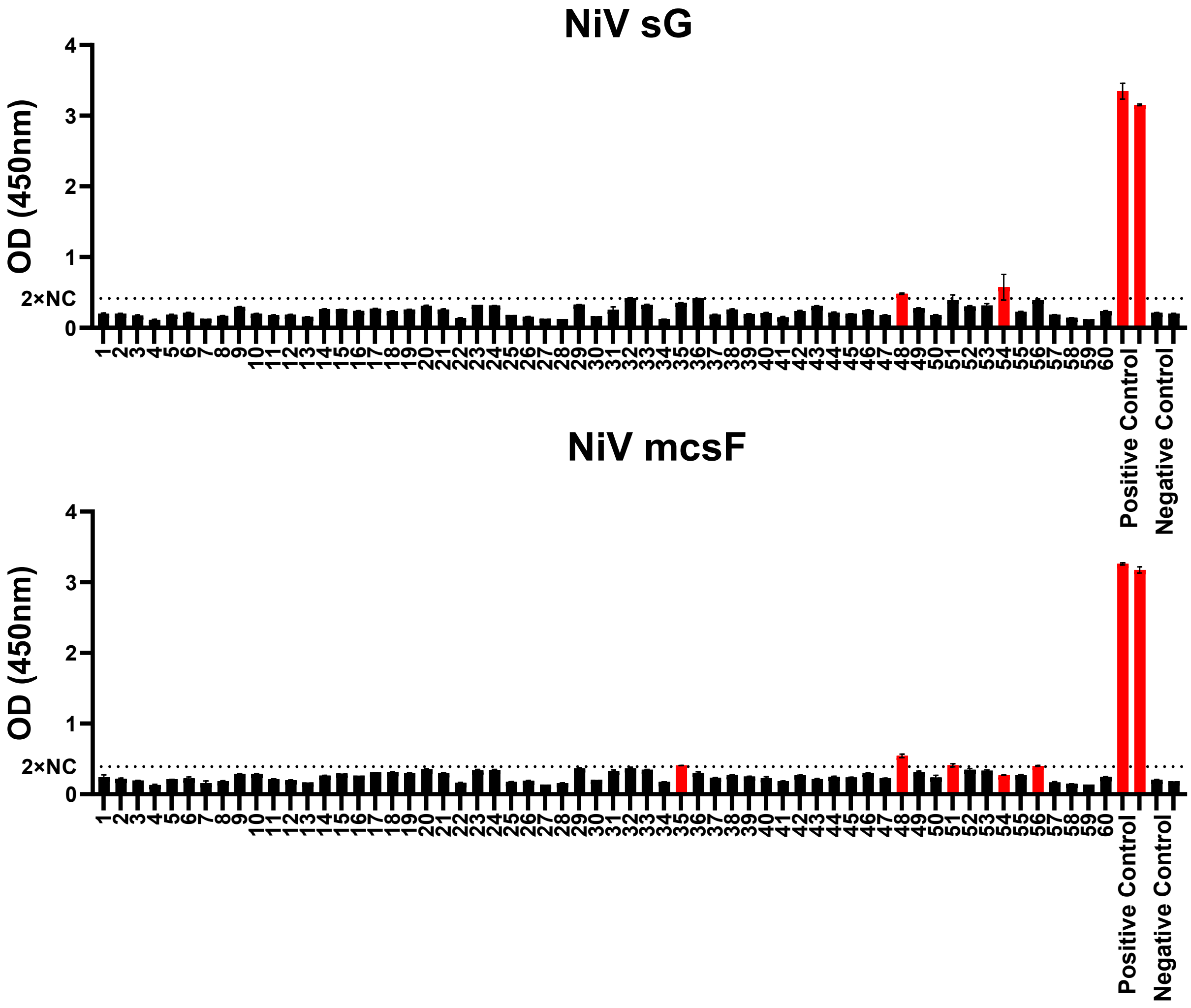

Supplementary Figure 9: Sixty indigenous breed pigs reared in backyard farming conditions in Bangladesh were pre-screened for NiV sG and mcsF reactive antibodies by ELISA. Positive samples (in red) were classed as those with a mean optical density (OD; 450 nm) reading twice the mean OD of negative control sera (dashed horizontal line). Positive control sera were from animals immunized with NiV sG or mcsF. Mean data for technical duplicates ± SD are shown for each serum.

Supplementary Table 1: Clinical scoring system for assessing vaccine reactogenicity in pigs. For the first vaccine immunogenicity study in pigs, the clinical score system comprised the below parameters measured daily from 0 – 41 days post-vaccination.

Parameter		Criteria	Score
1	Alertness	Attentive (curious, alert)	0
		Slightly reduced – hesitant, disinterested	1
		Inactive, gets up only when stimulated, lies down again	2
		Recumbent, won't get up when stimulated	3
2	Body shape/posture	Relaxed, full stomach, 'round' body	0
		Hunched back, empty stomach, thinned body muscles	2
		Flaccid or hunched and rigid body, emaciated, ribs & backbone showing	3
3	Coat/skin at injection site	Evenly light pink skin, hair coat flat	0
		Reddened or pale skin, slightly raised at injection site	1
		Swelling at injection site	2
		Bruising and/or painful swelling at injection site (either to touch or affecting movement)	3
4	Appetite	Greedy, hungry, all food finished	0
		Eats slowly when fed, some food remaining	1
		Does not eat when fed but tastes food. Food only partially eaten.	2
		Shows no interest in food, nothing eaten/drunk.	3
5	Rectal temperature	37.0 – 38.9 °C	0
		39.0 – 39.9 °C or less than 37°C	1
		40.0 – 40.9 °C	2
		41.0 °C or above	3
